# Targeting the Complement–Microglia Axis for Neuroprotection in Pediatric Epilepsy

**DOI:** 10.3390/biomedicines14081788

**Published:** 2026-08-08

**Authors:** Marah Karayanni, Nikolaos Mitsoudis, Maria Vanakliotou, Christos Bakirtzis, Evangelia Kesidou, Eleni Polyzoidou, Ekaterini Liana

**Affiliations:** 1 Department of Pediatrics, “Mamatsio” General Hospital of Kozani, 50100 Kozani, Greece; marahkarayanni@gmail.com (M.K.); mariavanakliotou@gmail.com (M.V.); stlkaterina@gmail.com (E.L.); 2 2nd Department of Neurology, Aristotle University of Thessaloniki, 54636 Thessaloniki, Greece; nickmitsoudis@gmail.com; 3 Laboratory of Experimental Neurology and Neuroimmunology, 2nd Department of Neurology, Aristotle University of Thessaloniki, 54636 Thessaloniki, Greece; kesidoue@auth.gr (E.K.); elpolyz@auth.gr (E.P.); 4 Laboratory of Physiology, Faculty of Medicine, Aristotle University of Thessaloniki, 54124 Thessaloniki, Greece

**Keywords:** pediatric epilepsy, developmental and epileptic encephalopathies, complement cascade, synaptic pruning, neuroprotection

## Abstract

Neuroprotection in childhood developmental and epileptic encephalopathies may require approaches, distinct from adult brain injury models of neuroprotection, with a primary focus on preservation of synaptic density rather than prevention of cellular necrosis. There is growing evidence to indicate early-life seizures activate complement cascade proteins C1q and C3. Subsequently, localized microglia may excessively phagocytose structurally intact synaptic neurons disrupting normal brain maturation. This review incorporates kinetic models of neuro-immune interactions based on human histopathology from epileptogenic tissues and quantitative neuro-immune biomarkers to provide suggestions that complement-mediated synaptic pruning may contribute to structural network disruption and cognitive decline in pediatric epilepsy. While standard anti-seizure medications effectively stabilize electrical activity, they do not mitigate underlying neuro-inflammatory responses. Consequently, targeted pharmacological inhibition of the complement microglia axis may provide a potential disease modifying strategy to protect developing neural circuits. The translational feasibility of using targeted complement inhibitors should be evaluated addressing critical challenges such as central nervous system drug delivery across the blood–brain barrier, immunosuppression management and the application of non-invasive biomarkers to define the precise therapeutic window for intervention.

## 1. Introduction

The concept of neuroprotection in developmental and epileptic encephalopathies (DEEs) requires a shift from traditional adult models of brain injury. Historically, neuroprotection has focused on preventing neuronal apoptosis and necrosis following acute insults like cerebral ischemia [1]. However, this focus on simply preventing cell death may be inadequate in pediatric epilepsy, as it could underrepresent how the infant brain is actively building its neural networks. In DEEs, long-term cognitive decline is rarely driven by widespread cell death alone; rather, it may be strongly influenced by excessive synaptic loss [2]. Early in life, the child’s developing brain rapidly constructs new connections through synapse formation and then eliminates non-essential ones through experience-driven synaptic elimination. This represents an important time of neurodevelopment when resident brain cells use complement proteins to define and organize the architecture of the brain as well as construct functional neural circuitry [3]. The disruption caused by infantile seizure activity may lead the complement system to identify healthy synapses for destruction.

Pediatric epilepsy is thought to lead to abnormal structural and functional alterations in the developing brain through a variety of mechanisms including disruption of networks, loss of neurons/synaptic contacts and changes in developmental pathways [2]. Since the cortex is still forming its connections during infancy, recurring/acute seizures may damage existing brain tissue and disrupt normal brain development. Furthermore, early life seizures may lead to microglial activation. Once activated, these cells can trigger the complement cascade and thus remove too many healthy synaptic connections that were needed for proper function [4].

Neuroprotection in this developing brain could go beyond cellular survival and the timely suppression of electrical seizures, to include the preservation of synaptic density. Therefore, this review aims to synthesize current pre-clinical, histological and clinical biomarker data demonstrating that complement-mediated microglial phagocytosis may play a central role in the neurodevelopmental abnormalities of minors with DEEs. Furthermore, this review will evaluate the feasibility and clinical potential of targeting the complement–microglia axis as a disease-modifying neuroprotective strategy.

## 2. Pre-Clinical Evidence of Aberrant Synaptic Pruning

In a healthy, developing central nervous system (CNS), neural circuits are continuously refined to establish functional networks. This refinement relies on resident microglia, which regulate synaptic density through a process known as synaptic pruning [3]. The physiological elimination of less active synapses is guided by the classical complement cascade, an immune pathway utilized during early neurodevelopment.

During growth, the complement system’s protein components, including C1q and C3, function as biological markers for the processes described above [4]. Typically, C1q is localized to specific synapses during normal development and acts as the first recognition molecule. The presence of C1q on a particular synapse signals a localized series of events resulting in the cleavage of C3. Following complement cascade activation, C3 is cleaved to deposit C3b on target synapses. Importantly, this deposited C3b is subsequently cleaved by regulatory proteins into the inactive fragment iC3b. Microglial-mediated synaptic engulfment is then executed through the specific recognition of this synapse-associated iC3b by the microglial complement receptor 3 (CR3) [5]. Following attachment, the microglial cell engulfs and degrades the targeted synapse. As such, this controlled removal of the developing synapse promotes an optimal ratio of excitatory to inhibitory neuronal connectivity.

While it is normally required for developmental processes to undergo some degree of complement-mediated synaptic elimination, research has shown through in vivo studies utilizing acute chemoconvulsant rodent models of acquired status epilepticus that abnormal activation of this process may be triggered by repeated episodes of early life seizure activity. Schartz et al., using postnatal day 14 (P14) and postnatal day 21 (P21) rodent models of status epilepticus caused by either lithium–pilocarpine or kainate, demonstrated that the interactions between the immune/neuro-physiological systems within the brain and its synapses are altered due to prolonged epileptiform activity [6].

The most consistent finding in animal models after seizures have been induced, is an increased expression of the complement system, specifically the protein products of C1q and C3, which indirectly contribute to the formation of the membrane attack complex (MAC). This increase is mainly observed within the hippocampus and those areas of the brain that exhibit abnormal electrical activity associated with seizures [6]. Instead of focusing on tagging fewer active connections, status epilepticus disrupts normal neuronal function so extensively that it may cause the complement system to deposit its protein products on functioning, firing neurons [7].

Consequently, localized microglia detect this increased density of C3b tags and transition to a reactive, amoeboid morphology. Rather than performing targeted maintenance, they begin to excessively engulf structurally intact dendritic spines [7]. Specifically, experimental data strongly demonstrates that complement-deficient (C3-/-) knockout mice subjected to severe status epilepticus exhibit a statistically significant reduction in histochemical neurodegeneration and pathological microglia–astrocyte interaction compared to wild-type mice [8]. Furthermore, complementary studies utilizing similar C3-deficient models have shown that blocking this cascade also preserves synaptic density and protects against the severe recognition memory deficits typically induced by prolonged seizures [9].

This dependence on developmental timing helps explain why adult-onset seizures rarely produce the same widespread cognitive decline observed in pediatric encephalopathies. During infancy and early childhood, the complement–microglia axis is naturally upregulated across the cortex to facilitate physiological synaptic pruning [10]. Consequently, epileptiform activity during this period alters a system that is already active, shifting physiological refinement toward structural degradation. In contrast, the healthy adult brain maintains a largely quiescent complement system. In the adult CNS, complement-mediated pruning is typically restricted to discrete regions that retain high synaptic turnover and neurogenesis, such as the dentate gyrus of the hippocampus [11]. Therefore, while acute adult seizures can trigger aberrant pruning in these specific regions, contributing to the localized memory deficits characteristic of adult temporal lobe epilepsy, they do not encounter the globally distributed, active neuro-immune infrastructure that facilitates the broader encephalopathy seen in the developing pediatric brain.

## 3. Histopathological and Molecular Findings in Human Epileptogenic Tissue

In addition to providing evidence regarding mechanisms that generate seizures in experimental models, resection specimens obtained during surgery provide an opportunity to study directly the biologic processes involved in generating seizures in patients with drug resistant focal epilepsy; including those with temporal lobe epilepsy (TLE) and focal cortical dysplasia (FCD), a frequent cause of medically intractable epilepsy in children [12]. FCD is characterized by localized areas of abnormal brain development paired with persistent tissue inflammation [13]. Therefore, excised specimens represent a valuable resource for examining the local cellular microenvironment of affected areas to determine whether the immune mediated pathways seen in experimental models also occur in clinical samples.

When interpreting human histopathological data, it is critical to stratify the evidence by patient age and underlying etiology. Currently, much of the direct tissue evidence for complement activation in human epilepsy originates from adult or mixed-age surgical cohorts. For instance, foundational studies demonstrating C1q and C3 upregulation in FCD specimens utilize tissue from predominantly adult subjects (e.g., mean surgical age of approximately 31 years) [14]. While these findings serve as mechanistic proof-of-concept that the complement–microglia axis drives aberrant synaptic reorganization in human drug-resistant epilepsy, they must be cautiously extrapolated when discussing exclusively pediatric DEE pathology.

Recent examinations of human epileptogenic lesions, including FCD, reveal an altered cellular ecosystem characterized by chronic inflammation and glial cell activation. For example, tissue studies have documented a significant accumulation of microglia, which gather around dysplastic neurons and blood vessels to create a localized inflammatory environment [15]. Rather than functioning normally, these microglial populations, alongside reactive astrocytes, show a coordinated activation of inflammatory pathways.

Importantly, some components of the classical complement cascade have been demonstrated to be significantly altered in those human samples. Surgically removed tissue samples from patients who suffer from refractory epilepsy often present increased levels of the proteins of the complement component C1q and downstream activation products such as iC3b, which is a fragment of the protein C3 [14]. Previous research has also demonstrated that there is an increase in the expression of both C1q and C3 in resected hippocampal tissue from patients with temporal lobe epilepsy; therefore, this immune system pathway may be linked to areas experiencing neuronal loss and severe network alterations [12].

Quantitative molecular profiling of human epileptogenic neocortex has revealed a statistically significant upregulation of C1q, C3 and downstream phagocytosis signaling molecules, as compared to healthy autopsy controls [14]. In addition, studies on Rasmussen’s Encephalitis—a serious type of pediatric epilepsy that is characterized by progressively worsening cortical atrophy as it moves to different parts of one hemisphere of the brain—have demonstrated that cerebrospinal fluid (CSF) and tissue analysis are indicative of increased levels of complement proteins such as iC3b, C5a and soluble MAC [16]. The dysregulation of complement control mechanisms in Rasmussen’s syndrome directly correlates with worse seizure profiles, strengthening the clinical link between complement activation and progressive neurodegeneration in distinct pediatric populations [17].

**Figure 1:**
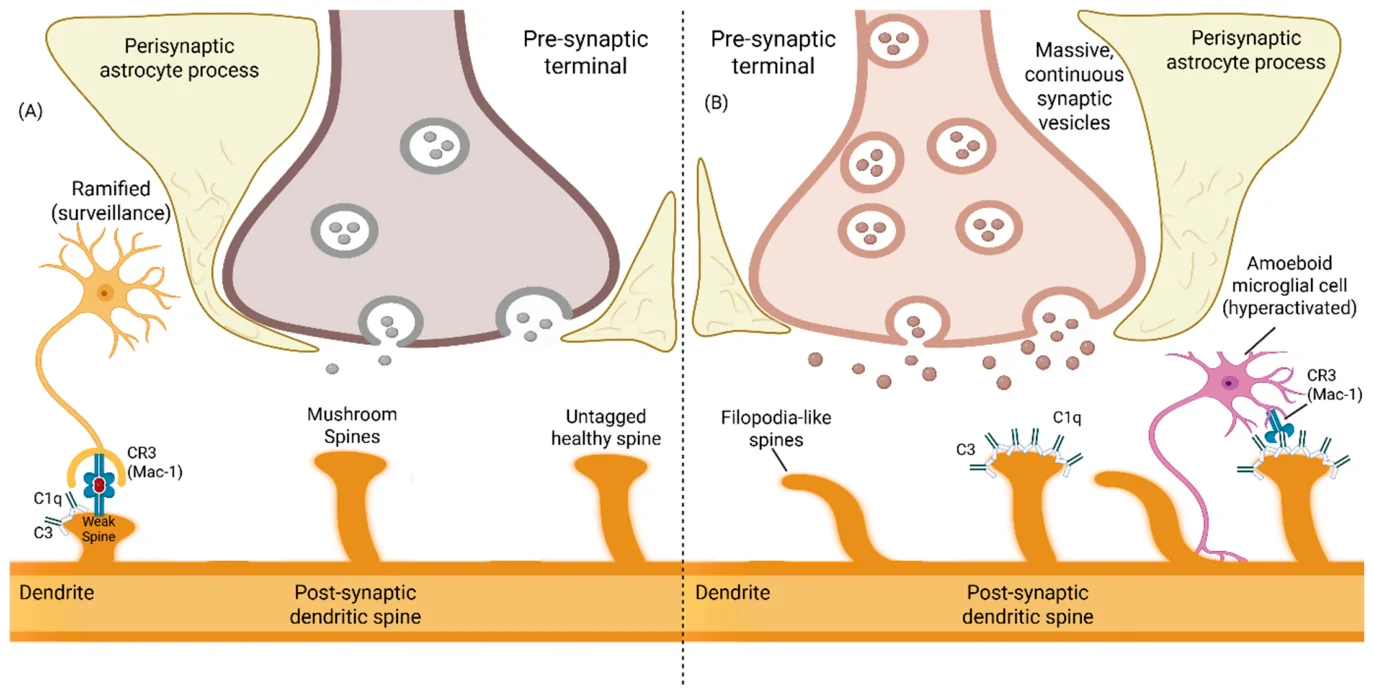
Physiological vs. Pathological Complement-Mediated Synaptic Pruning. (**A**) Physiological Surveillance: In a healthy developing cortex, ramified microglia continuously surveil the synaptic environment. The classical complement cascade selectively opsonizes less active or “weak” dendritic spines with C1q and C3 (C1q/C3-tagged). Microglial CR3 (Mac-1) receptors recognize these tags, facilitating the targeted, experience-dependent elimination of specific connections to maintain an optimal network architecture. (**B**) Seizure-Induced Aberrant Pruning: Following severe epileptiform activity, characterized by massive presynaptic vesicle release, the neuro-immune axis is aberrantly activated. Microglia undergo a morphological shift into a hyperactivated, amoeboid state. Concurrently, the complement system erroneously deposits high densities of C3 (and upstream C1q) onto structurally intact, healthy spines, driving them toward a degraded, filopodia-like state. Reactive microglia subsequently engulf these viable synapses via CR3-mediated phagocytosis, resulting in the pathological network disruption and structural degradation characteristic of developmental and epileptic encephalopathies.

Furthermore, histopathologic examination of human neocortical tissue has documented an immunologically mediated form of pruning as evidenced by physical alterations in the tissue (Figure 1). Studies confirm that C1q is localized to both microglia and neuronal dendrites in human tissue associated with epilepsy, and that there are considerable numbers of microglial processes physically interacting with these dendrites [14]. These characteristics are especially pronounced in Type II FCD, where histopathological evidence obtained from biopsies shows elevated levels of both C1q and C3-fragments found directly within the dysplastic lesions and around reactive astrocytes. Classical complement pathway activation and microglial reactivity are significantly higher than those observed in normal control tissue. This activation is highly correlated to areas of significant dendritic spine loss and appears to be driving a microglia-mediated destruction of the dendritic spines [18].

Combined, these observations of microglial accumulation, local complement deposition, and direct microglial–dendritic interactions provide evidence that the aberrant immune signaling pathway actively occurs in human refractory epilepsy, directly contributing to the synaptic reorganization seen in these conditions.

While these histopathological findings confirm an altered neuro-immune environment, they must be interpreted with caution. Human surgical tissue studies are inherently cross-sectional and affected by severe selection bias, representing only end-stage pathology in highly pharmacoresistant patients. Consequently, this observational data cannot definitively determine whether complement activation is a primary driver of the pathology or a secondary consequence of intractable seizures, nor can it confirm that this specific pathway accounts for cognitive decline across the broader spectrum of pediatric DEEs.

## 4. Clinical Biomarkers and Cognitive Correlates

To better understand the biological processes occurring in DEEs, researchers analyze CSF and blood serum from pediatric patients to identify specific markers of neuroinflammation. However, it is necessary to stratify these biomarker profiles, as they vary significantly depending on the specific epilepsy syndrome and etiology. For example, clinical data indicate that children with acute, highly inflammatory acquired early-onset epilepsy syndromes, including febrile infection-related epilepsy syndrome (FIRES), Lennox–Gastaut syndrome and prolonged status epilepticus, have an altered immune profile compared to healthy controls [19]. Conversely, in genetic DEEs, such as Dravet syndrome (a genetic disorder that causes a form of DEE due to mutation of the gene for the Na+ channel alpha subunit, SCN1A), the inflammatory profile is often more chronic, where microglia become very activated in response to this disease process and there is also a significant up-regulation of both complement and Toll-like receptors (TLRs) that further enhance or cause the hyper-excitability of neurons [20]. Furthermore, during severe episodes like new-onset refractory status epilepticus (NORSE) and FIRES, CSF interleukin -6 (IL-6) levels frequently surge to pathological levels (e.g., >110.0 pg/mL), vastly exceeding the normal physiological baseline of ≤7.0 pg/mL [19].

In contrast to severe encephalopathies, there is currently a lack of substantial evidence indicating significant complement activation in idiopathic generalized epilepsies (IGEs), such as childhood absence epilepsy [21]. IGEs are primarily driven by functional thalamocortical network alterations, often linked to inherited channelopathies, without the prominent neuroinflammation or microglial hyperactivation characteristic of DEEs [22]. This absence of widespread complement-mediated synaptic pruning aligns with the typical clinical trajectory of IGEs, which often feature preserved baseline cognition, a lack of progressive structural degradation, and age-dependent remission [23].

Beyond general inflammation, proteins involved in the classical complement cascade are also elevated in epileptogenic networks; the complement cascade is activated specifically through C1q and C3 in brain tissue from patients with temporal lobe epilepsy [24]. Genetic and proteomic studies have also confirmed that the gene and protein expression of C3 was increased in both pediatric and adult patients who suffer from pharmacoresistant mesial temporal lobe epilepsy associated with hippocampal sclerosis [12]. Since these proteins tag synapses for elimination by microglia, their presence at such a level during the period of epileptogenesis suggests that this pathway remains active.

As a direct consequence of this excessive neuro-immune activation, physical degradation of the neural network occurs, which may be tracked using structural biomarkers. Neurofilament Light Chain (NfL) is a primary structural marker in addition to the pro-inflammatory cytokines. The elevation in this biomarker in both CSF and serum in drug resistant epilepsy patients can be used as an indicator for potential neurological damage and disruptions in the blood–brain barrier (BBB) [25]. Research indicates that there is a statistically significant correlation between the length of time that a patient experiences status epilepticus and the concentration of NfLs in their serum. This analysis indicates that as seizures persist longer than expected they are associated with an neuroaxonal damage that correlates with the time of seizure activity [26].

The identification of these biomarkers may be beneficial as their level corresponds directly to an individual’s clinical symptomatology [27]. For example, high levels of NfL correlate well with the duration of status epilepticus and the presence of refractory epilepsy, offering a metric for short-term functional outcomes [26].

In addition, by determining the amounts of various complement proteins, researchers have been able to provide some insight into why there are electrophysiological and cognitive difficulties in children who suffer from seizures due to encephalopathy. Experimental data on temporal lobe epilepsy showed that if C1q was over-produced and then converted into iC3b, both were related to an increase in seizure frequency and a larger degree of cognitive impairment [7]. The activation of the C1q-C3 signaling pathway caused by status epilepticus continued until the child experienced additional seizures; hence, the amount of activation of the C1q-C3 transduction cascade was directly proportional to the number of current seizures [6].

## 5. Translating Mechanism to Therapy: The Feasibility of Complement Inhibition

### 5.1. The Synaptic Gap in Current Care

Current anti-seizure medications are the standard treatment for pediatric epilepsy [28]. These drugs work primarily by altering ion channels and neurotransmitter receptors. Their main function is to stabilize electrical activity in the brain. By doing this, they successfully reduce the frequency of seizures for many patients. While standard anti-seizure medication is important for preventing further acute seizure activity from occurring and preventing early excitotoxic necrosis, it has limited potential to modify an underlying disease process [29].

A broader evaluation of clinical and experimental literature supports the consensus that current ASMs act primarily as symptomatic treatments rather than true antiepileptogenic agents [30]. Longitudinal clinical studies have shown that despite the introduction of numerous new ASMs over the past several decades, the overall proportion of patients who develop drug-resistant epilepsy has remained largely unchanged, and cognitive comorbidities may still progress even when clinical seizures are adequately controlled [31]. Furthermore, in preclinical models of acquired epilepsy, administering standard ASMs (such as phenobarbital or levetiracetam) during or immediately after status epilepticus generally suppresses acute convulsions but does not consistently prevent the subsequent development of spontaneous recurrent seizures or chronic microglial activation [32]. These observations suggest that while stabilizing ion channels is essential for acute symptom management, it may be insufficient on its own to arrest the structural and immunological alterations driving DEEs [33].

To fully understand this therapeutic gap, it is necessary to distinguish between complement activation as a direct consequence of seizures versus an independent pathological mechanism. Evidence suggests a biphasic relationship: initially, severe epileptiform activity acts as the acute trigger for complement upregulation, meaning that early elevations in neuro-inflammatory biomarkers closely reflect the severity of the initial seizure burden [26]. However, once activated, the complement–microglia axis can transition into a self-perpetuating, independent cycle of chronic inflammation [34]. While standard ASMs may reduce further acute electrical triggers, they lack the specific pharmacological mechanisms required to directly inhibit this established neuro-immune loop. Consequently, this smoldering inflammatory state can persist even when clinical seizures are suppressed, continuing to drive aberrant synaptic pruning and progressive cognitive decline [35]. This persistence highlights the necessity of developing targeted immunomodulators that act directly on the complement cascade to complement the electrical stabilization provided by ASMs.

The fact that current AEDs cannot manage the secondary network disruptions caused by inflammatory activity creates a gap in the treatment of modern epilepsy. The clinician can control clinically evident seizures with existing treatments; however, the underlying cause of the pathology or disease process (i.e., the activation of the immune system) may be still present and uncontrolled [36]. As such, further research into complement inhibition as an adjunctive/synergistic therapy that halts secondary network disruptions while maintaining the effectiveness of electrical control is needed.

### 5.2. Emerging Pharmacological Targets

While current epilepsy treatments focus on arresting epileptiform activity, other neuroimmune disciplines are actively investigating targeted immunomodulatory agents to selectively inhibit the complement cascade. In fact, complement inhibitors are already used or under investigation for the treatment of several immune mediated neurological diseases [37]. In this context, two agents, ANX005 (anti-C1q treatment) and pegcetacoplan (anti-C3 drug), may be worth further investigation for their potential use in cases with severe pediatric epilepsy. While there are other established complement inhibitors available to treat generalized inflammation such as anti-C5 therapy for generalized Myasthenia Gravis and Neuromyelitis Optica Spectrum Disorder, directly targeting C1q and/or C3 would represent the best way to specifically inhibit microglia mediated synaptic pruning in the brain.

Importantly, neither ANX005 nor pegcetacoplan has demonstrated efficacy in pediatric epilepsy or the prevention of seizure-associated synaptic pruning. While these agents establish the general feasibility and safety of systemic complement inhibition in humans, their potential to halt synaptic degradation in DEEs remains untested and requires rigorous preclinical evaluation before human trials can be considered.

ANX005 is an investigational monoclonal antibody. It is designed to bind and neutralize C1q, which is the protein that starts the classical complement cascade [37]. Following systemic administration, ANX005 demonstrates CNS penetrance, effectively neutralizing C1q and attenuating downstream complement activation within the CNS. It has recently been evaluated in clinical trials for complement-mediated neurological conditions, including Huntington’s disease [38] and Guillain-Barré syndrome [39]. In these human trials, the drug was generally well-tolerated and demonstrated full, durable C1q target engagement in both serum and CSF, validating its safety profile and its ability to halt complement activation without significantly increasing the risk of severe infections [38]. By neutralizing C1q, therapies like ANX005 could potentially prevent the immune system from mistakenly tagging healthy synapses.

**Figure 2:**
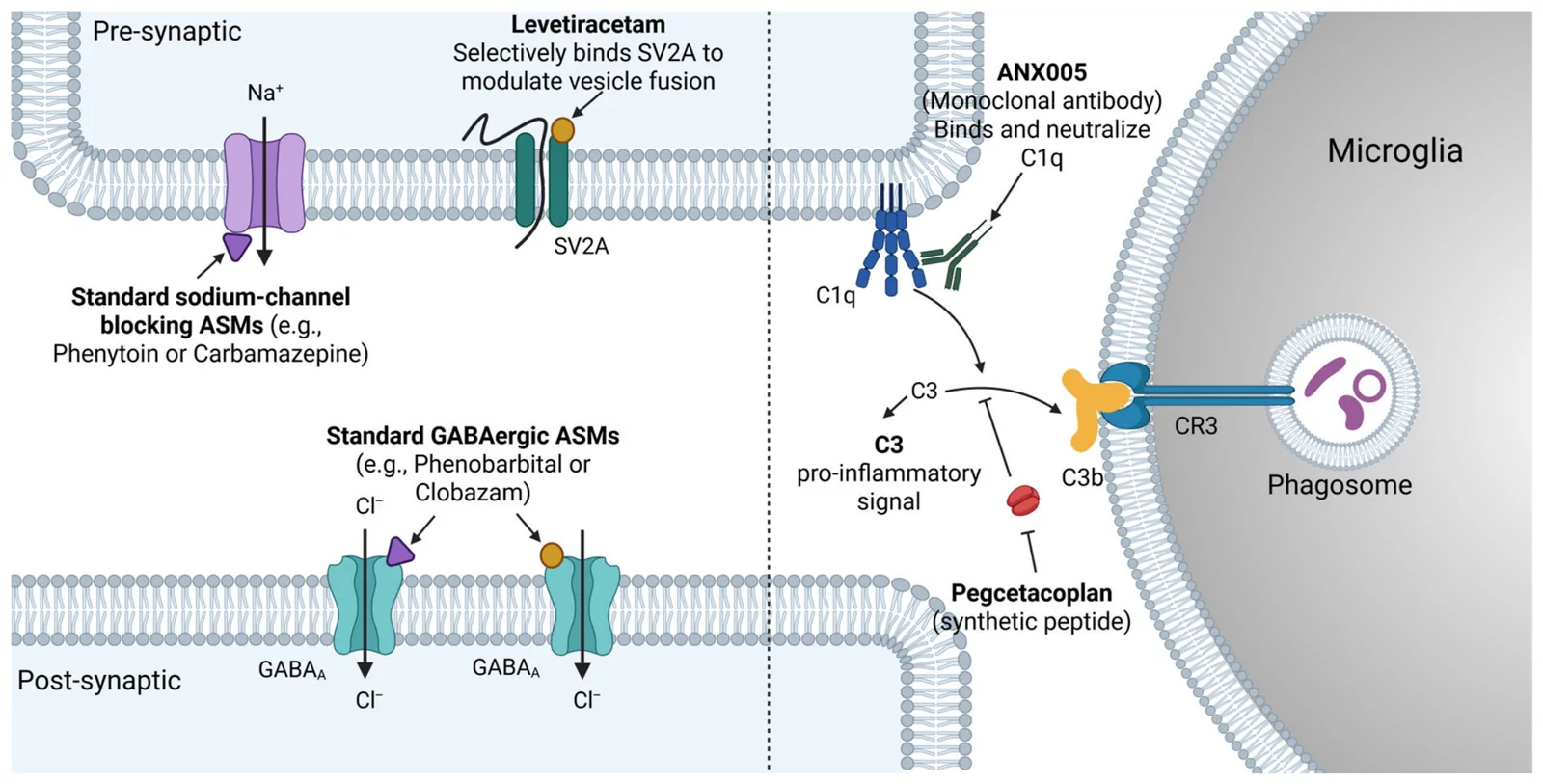
Bridging the Therapeutic Gap: Electrophysiological vs. NeuroImmune Pharmacological Targets. The diagram illustrates the distinct mechanisms of action between standard anti-seizure medications (ASMs) and emerging complement-targeted therapies. (**Left Panel**): Conventional ASMs achieve symptomatic seizure control by stabilizing electrical networks. Key mechanisms include sodium-channel blockers (e.g., phenytoin, carbamazepine) that limit presynaptic depolarization, levetiracetam modulating SV2A to attenuate neurotransmitter release, and GABAergic agents (e.g., phenobarbital, clobazam) that enhance postsynaptic inhibitory chloride (Cl^−^) influx. While effective for acute electrical suppression, these agents do not mitigate underlying neuro-immune cascades. (**Right Panel**): Targeted immunomodulatory interventions aim to halt aberrant synaptic pruning by inhibiting specific nodes of the classical complement cascade. ANX005, an investigational monoclonal antibody, directly binds and neutralizes C1q, preventing cascade initiation. Downstream, the synthetic peptide pegcetacoplan inhibits the cleavage of C3, preventing the release of the pro-inflammatory C3a signal and the deposition of C3b. By blocking C3b opsonization, these targeted therapies prevent the activation of microglial CR3 receptors, thereby shielding viable synapses from pathological phagocytosis.

Pegcetacoplan acts in the classical complement pathway by specifically inhibiting C3 cleavage through direct binding to the C3 molecule using a highly selective and potent peptide [40]. By doing so, this action should inhibit the breakdown of C3 into the active fragments that are required for the process of classical complement activation. Pegcetacoplan has been approved for clinical use by the U.S. Food & Drug Administration (FDA) for certain diseases including Paroxysmal Nocturnal Hemoglobinuria; a rare blood disorder [41]. and geographic atrophy; a disease associated with age-related macular degeneration and related to excessive activity of the classical complement pathway. Pegcetacoplan can be considered to potentially prevent or reduce the extent of the molecular tagging of target cells in the form of C3b fragments which are produced during classical complement activation [42]. As shown in Figure 2, targeted inhibition of C3 function in young mice results in reduced microglial CR3-mediated phagocytic activity on aberrantly tagged synaptic surfaces containing C3b. Consequently, critical neural circuits that would otherwise have been destroyed were preserved.

To further optimize the delivery of these large-molecule agents into the CNS, the recent structural and functional characterization of meningeal lymphatic vessels and the brain’s glymphatic system provides a promising avenue [43]. These perivascular networks facilitate the bulk flow of cerebrospinal fluid directly into the brain interstitial space. Consequently, researchers are investigating non-invasive routes, such as targeted intranasal delivery, to leverage this glymphatic architecture for CNS drug transport [43]. Utilizing these physiological fluid channels could theoretically allow targeted complement inhibitors to bypass the blood–brain barrier and achieve widespread parenchymal distribution, maximizing target engagement at epileptogenic synapses while minimizing systemic exposure.

### 5.3. Immunosuppression and Safety

The complement proteins C1q and C3 do more than just refine connections in the brain. They are vital parts of the body’s general immune defense [44]. This pathway helps the body clear common pathogens and fight off everyday infections. Blocking this system in pediatric patients requires careful thought. Children are still building their natural immunity and a suppressed complement pathway might increase a child’s risk for infections [45]. Fortunately, there exist established treatment guidelines in medicine to assist with managing many of the same risks that were discussed. In fact, when a patient is taking an existing complement inhibitor for another health condition they will almost always be under a protocol for the safe administration of their medication. This includes receiving prophylaxis prior to initiation on this type of medication. The medications given as part of this process are designed to provide protection against certain encapsulated pathogens, including meningococcus and pneumococcus [46]. In some cases, physicians may also prescribe a daily preventive antibiotic to add an extra layer of protection and/or meningococcal vaccination is mandatory before starting complement inhibitors. By carefully following these established clinical protocols, it might be possible to safely manage the immune risks.

### 5.4. The Therapeutic Window

Finding the optimal time to initiate immune-modulating treatments in pediatric epilepsy remains a complex translational challenge. While evidence indicates that aberrant synaptic loss is a secondary, delayed mechanism occurring after the initial electrical insult, the precise post-seizure kinetics of complement-mediated pruning in DEEs remain to be definitively established. Pre-clinical models have clearly demonstrated significant complement activation and synaptic loss during the sub-acute and chronic phases (e.g., 2 to 5 weeks) following status epilepticus [11]. Furthermore, cross-sectional human tissue studies confirm the presence of complement components in epileptogenic networks [14]. However, because human studies cannot establish post-seizure kinetics, the exact temporal onset of C1q/C3 synaptic tagging and the subsequent transition to active microglial phagocytosis during the acute phase require further longitudinal investigation. Consequently, while intervening before irreversible structural clearance commences is theoretically essential, defining the precise therapeutic window remains a critical gap in the field. To guide future dynamic biomarker studies, we propose a theoretical framework illustrating the hypothesized sequence of neuro-immune events (Table 1).

Moreover, we need to consider the fact that aberrant synaptic pruning in DEEs occurs as an episodic event due to a large-scale seizure, but pathological interictal epileptiform discharge (IEF), i.e., continuous subclinical firing patterns as observed through electroencephalography (EEG) between discrete seizures, maintains the neural network in a heightened state of distress at all times [47]. As such, this chronic, low-grade activation of microglial cells with the resultant complement system triggering is most likely responsible for maintaining a prolonged smoldering activation of the complement/microglia axis as opposed to an acute response [48]. In other words, while seizures provide us with a distinct time frame of approximately 48 h to potentially intervene to stop massive structural removal from occurring, the continuous nature of IEFs indicates to that long term, sustained modulation of the immune system via complement hyperactivation would likely be needed to slow or cease synaptic loss and subsequent progression towards a marked decrease in cognitive function [36].

However, pinpointing this exact therapeutic window is highly challenging. During early childhood, the brain naturally relies on regular synaptic pruning to grow, learn, and refine its circuits [3]. Thus, if clinicians were to inhibit the complement system too soon or for too long, they would likely interfere with normal and healthy brain development. On the other hand, because of an active immune response later than optimal, some connections which are crucial may have been eliminated. In such cases, both the structural loss of these connections and the associated cognitive deficits may be permanent.

To safely implement these therapies, clinical trials may benefit from relying on validated, practical biomarkers. While baseline and post-intervention analysis of CSF provides critical data on complement proteins like C1q and C3, continuous CSF sampling may be ethically and clinically unfeasible in pediatric cohorts [49].

In addition to fluid biomarkers, researchers may use advanced imaging to monitor the brain’s physical state and therefore establish trial endpoints that combine discrete, targeted CSF evaluations with non-invasive surrogate markers [50]. For example, by implementing serial advanced diffusion tensor imaging (DTI), longitudinal mapping of microstructural network integrity and progressive white matter degeneration may be quantified, without having to perform repeated lumbar puncture procedures on pediatric patients [51]. It is imperative to develop a realistic, non-invasive biomarker profile for the purpose of determining an appropriate therapeutic window; assessing drug efficacy; and developing feasible inclusion criteria for pediatric neuroprotective clinical trials.

## 6. Limitations

While targeting the complement–microglia axis offers a compelling neuroprotective hypothesis, several translational considerations must be addressed before clinical application. A primary obstacle for complement inhibition in the CNS has been the belief that large-molecule therapeutics, such as monoclonal antibodies, must actively cross the intact BBB to achieve adequate penetrance [52]. In severe developmental and epileptic encephalopathies, prolonged seizures actively cause temporary, localized leaks in BBB tight junctions [53]. Therefore, rather than acting solely as an unpredictable barrier, this disease-altered state may naturally facilitate the entry of systemic immunomodulators directly into epileptogenic networks precisely when aberrant synaptic pruning is most aggressive. Nevertheless, to optimize target engagement during the chronic, interictal phases and avoid relying exclusively on acute inflammatory leaks, contemporary neuropharmacology is actively exploring next-generation delivery platforms, such as lipid nanoparticles, to transport these agents across the intact BBB [54].

Beyond delivering drugs, many other limitations exist for the biological/clinical monitoring of children. During early childhood, there are several fundamental mechanisms that depend on the developmentally regulated interaction of the complement system with microglial cells. For example, normal physiological synaptic pruning, as well as experience-dependent plasticity and cognitive maturation all require the presence of both a functioning complement system and microglial cells. Theoretically, chronic systemic inhibition of C1q or C3 at this developmental time point may result in disrupting this developmental pruning, potentially inducing neuropsychiatric phenotypes [24]. Additionally, since the repeated sampling of CSF from pediatric patients for dynamic titration of their immunomodulatory dosages would be extremely challenging, the use of highly sensitive and non-invasive in vivo biomarkers that can distinguish between active pathological phagocytic activity and normal developmental refinement may be required [55].

## 7. Conclusions

Pediatric DEEs are complex conditions in which neuroimmune dysregulation is increasingly recognized as a significant component of the underlying pathology. In these conditions, severe prolonged seizures can lead to an abnormal activation of the classical complement cascade that leads to microglia consumption (phagocytosis) of structurally healthy synapses. Since standard anti-seizure medications do not halt this ongoing neuroinflammation after an electrical insult, children with drug resistant profiles are at high risk for long-term structural degeneration of their brain networks and irreversible cognitive decline.

Therefore, protecting a developing child’s neurological function may require expanding beyond simply reducing the amount of electrical activity in the developing child’s brain. Targeted pharmacologic inhibition of specific components of the complement system (anti-complement treatments) such as C1q and C3 could offer potential disease modifying strategies to help preserve synaptic density in the developing child’s brain. However, there are several translational barriers including optimizing CNS drug delivery systems, managing the risks associated with systemic immunosuppression and validating biomarkers to determine optimal time frame for therapeutic intervention. It is essential to address these challenges, as bridging the gap between controlling seizures and providing potential neuroprotective effects, may ultimately allow mitigation of long-term deterioration of the cognitive trajectory in these vulnerable populations.

## Figures and Tables

**Table 1:** Theoretical Framework of Seizure-Induced Synaptic Pruning. Because exact post-seizure kinetics remain undefined in human DEEs, this table presents a hypothesized sequence of neuro-immune events. Identifying the exact temporal boundaries of these phases through longitudinal biomarker studies is critical for establishing a therapeutic window before active microglial phagocytosis (observed at sub-acute/chronic time points) drives irreversible cognitive decline.

Hypothesized Phase	Post-Insult Timeline	Biological Mechanism	Clinical Relevance
Acute /Early Initiation	Unknown (Requires Acute Longitudinal Profiling)	Initial seizure activity acts as the trigger. Potential early upregulation of complement precursors and neuro-inflammatory cytokines.	Standard anti-seizure medications stabilize electrical activity. Represents the theoretical opening of the window for immunomodulation.
Intermediate/Tagging	Days to Weeks (Target of Future Study)	Complement cascade processes C3. Pathological deposition of C3b/iC3b tags onto structurally viable, active synapses.	The critical inflection point for therapeutic intervention. Modulating the cascade here theoretically prevents tags from signaling microglia.
Sub-Acute/Chronic Phase	Weeks to Months (e.g., 2–5 weeks post-insult)	Microglia transition to an amoeboid state, recognizing iC3b via CR3 receptors, resulting in active structural engulfment [6].	Irreversible structural network clearance begins. Complement inhibition initiated at this late stage may be ineffective at rescuing lost cognitive function.

## Data Availability

No new data were created or analyzed in this study. Data sharing is not applicable to this article.

## Abbreviations

The following abbreviations are used in this manuscript:

Abbreviation	Definition
AEDs	Anti-Epileptic Drugs
ASMs	Anti-Seizure Medications
BBB	Blood–Brain Barrier
CNS	Central Nervous System
CSF	Cerebrospinal Fluid
DEEs	Developmental and Epileptic Encephalopathies
DTI	Diffusion Tensor Imaging
EEG	Electroencephalography
FCD	Focal Cortical Dysplasia
FIRES	Febrile Infection-Related Epilepsy Syndrome
IEF	Interictal Epileptiform Discharge
IL-6	Interleukin-6
MAC	Membrane Attack Complex
NfL	Neurofilament Light Chain
NORSE	New-Onset Refractory Status Epilepticus
P14/P21	Postnatal Day 14/Postnatal Day 21
TLE	Temporal Lobe Epilepsy
